# College and Hospital Notes

**Published:** 1899-12

**Authors:** 


### COLLEGE AND HOSPITAL NOTES.

THE private hospital formerly conducted at 304 Lafayette Avenue is again open for the reception of private patients. It is the intention of the management to admit only the best class of private patients and to give them the best of care and attention. The hospital has been newly refurnished and made into a first-class private hospital, with all modern improvements and conveniences, and called the Lafayette Hospital. There is no staff of physicians in attendance; each physician sending a case has full control of the patient. Mrs. Lillian C. Lacey, who is in charge, is an experienced nurse and a capable manager. A cordial invitation is extended the profession to inspect the hospital.

---

A NEW emergency hospital is projected in Buffalo to replace the present one, which was established in 1883 as an adjunct to the Buffalo Hospital of the Sisters of Charity. This latter has become inadequate to the present needs, hence the authorities represented by the Sisters propose to build a new one.

It is stated that the new hospital will be thoroughly equipped, roomy and perfected in every particular. It will be four stories high, and there will be room for more than 100 beds, besides accommodations for nurses and other help necessary to the institution. Great care will be given to hygienic arrangements; there will be light on four sides, and heating and ventilating facilities, of the most approved kind, will be introduced.

An elevator of sufficient size to admit patients upon a stretcher will be built and cases needing surgical aid will be carried in the elevator to the fourth floor, where the operating room will be located. There will be wards for general patients and separate wards for each of the railroads. Private rooms will also be provided for such as may require them.

At the rear of the hospital will be the stables, which will contain two ambulances under "quick hitch" regulations, the drivers' apartments being overhead. Neatly arranged grounds, with lawns, shrubbery, etc., will surround the building.

---

LADY RANDOLPH CHURCHILL is heading the American women's movement in London for fitting out a hospital ship for South African Service, and the American committee has already collected more than $40,000, after the Atlantic Transport Company has supplied the vessel. Mrs. James Brown Potter recently gave a concert and dramatic recital for the fund which realised a large sum. The entertainment was held at Claridge's Hotel and was called a thé chantant. A London despatch of November 18th, among other things said that the exquisitely decorated rooms were thronged with the exclusive set of fashionable people in brilliant attire, including the Prince of Wales, the Duke of Cambridge, Princess Christian, of Schleswig-Holstein, Princess Aribert, of Anhault and Princess Victoria of Schleswig-Holstein. The last three were accompanied by Mrs. Hopkins, of New York, the Duchess of Marlborough, Mr. and Mrs. Joseph H. Choate, Mrs. Ogden Goelet, Mrs. Arthur Paget, Lady Tweedmouth, Lady Lansdowne and a host of others.

The hospital ship is to be called The Maine in honored memory of our battleship, which was blown up by the Spaniards in Havana harbor, and something over £1,700 was added to the Maine fund as the result of the entertainment. A more charming and successful gathering has seldom been witnessed at any social function in London than was present at that of the American women in their effort to increase the Maine fund.

At Lady Randolph's request Mrs. Whitelaw Reid has already selected and sent forward American nurses for the ship. It is quite evident that war in London charities will be conducted on a large scale throughout the winter, and that all the arts and wiles of cadging will be devoted to patriotic use. Life has suddenly become real and earnest among the gilded youth and fashionable dawdlers in Vanity Fair.

---

It is announced that Niagara Falls is soon to be supplied with a maternity hospital. A recent dispatch says that Messrs. Orchard & Jorolemon, architects of that city, are engaged in preparing plans for a maternity hospital which is to be constructed and presented to the City of Niagara Falls by Benjamin F. Thurston, as a memorial of his wife, who died recently.

The hospital it is stated will be as well equipped and complete as any in the state. It will be located to the left of the Memorial Hospital, and will be of the colonial style of architecture. It will be divided into private and public wards.

---

At the annual meeting of the New York Skin and Cancer Hospital, at Second Avenue and Nineteenth Street, on November 17th, the following officers were elected: President, J. Cleveland Cady; vice-president, William C. Witter; secretary, Dr. L. Duncan Bulkley, and treasurer, Edward Winslow.

The report for the year shows remarkable growth in the work of the hospital. More than 3,000 cases have been treated, and 13,000 visits made in its out-patient department—a third more than ever before.

The field of this hospital is mainly among working people. An eczema that renders a teacher, saleswoman or waitress unacceptable on account of her appearance, or that destroys the sleep of an engineer or laboring man, ends self-support and independence. To such it holds out the only relief they are likely to find, for it is the only one in the country that makes a specialty of such maladies.

The hospital is dependent upon the public for support in doing its work of "helping people to help themselves."—*New York Tribune.*

